# Investigation into the Synthetic Strategies of Melamine-Based Porous Polymeric Materials: A Bibliometric Analysis

**DOI:** 10.3390/polym17070868

**Published:** 2025-03-24

**Authors:** Nazeeha S. Alkayal

**Affiliations:** Chemistry Department, Faculty of Science, King Abdulaziz University, P.O. Box 80203, Jeddah 21589, Saudi Arabia; nalkayal@kau.edu.sa

**Keywords:** melamine, porous organic polymer, synthetic strategies, bibliometric

## Abstract

Recent years have seen the rapid development of melamine-based porous organic polymers, a new category of porous material. This review paper comprehensively describes the progress and trends of melamine-based porous organic polymers by using bibliometric analysis. A total of 1397 publications published over the previous 15 years were extracted from the Web of Science Core Collection database. Also, cooperation between countries and affiliations and keyword co-occurrence were assessed with the aid of VOS viewer software 1.6.20. The findings suggested that this field of study is now rapidly evolving. From 41 articles in 2009 to 180 articles in 2022, the number of published articles has increased significantly. China was the most productive nation, publishing 863 articles with 61.78% contribution. The Chinese Academy of Sciences was the most productive organization, and Chinese author Qiang Li was the most productive individual. Keyword co-occurrence analysis identified major research hotspots, including the design of high-surface-area materials for adsorption, functionalization strategies to enhance materials’ performance, and novel synthesis routes for structural control. Furthermore, this review systematically categorizes synthetic strategies based on the linkage structures between melamine and other building blocks, providing insights into state-of-the-art advancements and future research directions in the field.

## 1. Introduction

Porous organic polymers (POPs) are a large class of materials with structures that show a high degree of crosslinking. They are made up of many pure organic building blocks that are bonded together by strong covalent bonds. The various building blocks that can be used to construct POP materials give them unique structural, functional, and attribute characteristics. POPs have garnered significant interest because of their distinctive properties, which include permanent porosity [1], large surface area, minimal density [2], and outstanding chemical/thermal stability [3]. Permanent porosity refers to the ability of these polymers to maintain their porous structure, even in the absence of guest molecules, distinguishing them from traditional porous materials that may collapse upon solvent removal. This intrinsic characteristic arises from the rigid and crosslinked nature of the polymer network, which prevents structural deformation and ensures continuous accessibility to the pores. Furthermore, POPs exhibit remarkable stability in almost all common solvents, including water, and at different pH levels [4]. POPs fall under a variety of network categories, such as covalent triazine frameworks (CTFs) [1], conjugated microporous polymers (CMPs) [5], hyper-crosslinked polymers (HCPs), and covalent organic frameworks (COFs) [6], etc. POPs are typically created by bottom-up chemical reactions, which provide the option to add different functionalized building blocks to alter the structure of the final product [7]. Over the years, a number of chemical reactions, including Suzuki cross-coupling, Schiff-base condensation, ionothermal method, Friedel–Crafts alkylation, Diazo-coupling, and Sonogashira reaction, have been identified and used to produce a variety of POPs materials [8,9,10,11,12]. There are a lot of potential applications that can be fulfilled by the diversity of preparation strategies and building blocks employed to build POP materials.

**Figure 1:**
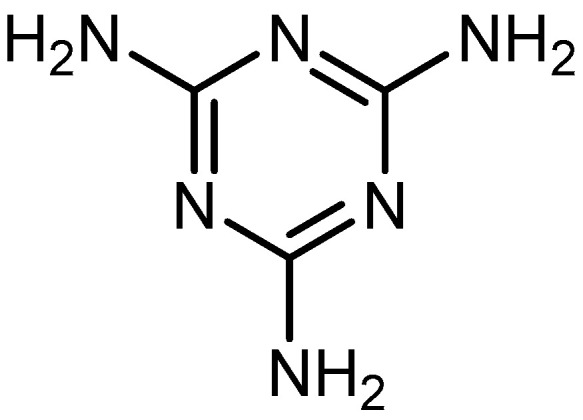
Melamine structure.

POPs formed from melamine (MA) monomers are intriguing materials because they can be synthesized from inexpensive building blocks and have a variety of uses in environmental treatment, gas absorption, and catalysis [10,11,13]. Melamine is a six-membered heterocyclic aromatic organic compound with the formula (C_3_H_6_N_6_), and is also known as 2,4,6-triamino-1,3,5-triazine (Figure 1). In terms of physical characteristics, it is a crystalline, white, odorless, and nontoxic substance. It is a cheap chemical that has a mass percentage of 66% N and is frequently used in coating, plastic, and pharmaceutical processes [12,14]. Furthermore, the triazine ring’s electron-donating effect on the attached primary amino groups gives melamine a significantly higher basicity [15]. Through π–π interactions, metal coordination, and hydrogen bonding, melamine can react with other molecules [14]. It is therefore a great choice as the precursor material for the synthesis of N-rich polymers. Under proper polymerization reaction conditions, MA may interact with carboxylic acids, dianhydrides, nitro compounds, and aldehydes to produce unique melamine-based polymers such as polyamide, polyimide, azo-linkage, and polyaminal networks, respectively [6,16]. Consequently, we are interested in melamine-based polymer networks as a class of porous organic polymers due to their ease of synthesis, large specific surface areas, adjustable basic functionalities and low-cost monomers [17].

The following features of the melamine-based porous organic polymeric materials are derived from the collected literature in this review article and are listed below:

1. Melamine-based POPs are synthesized under various kinds of experimental circumstances, involving reactions conducted at ambient temperatures.
2. They are prepared by many molecular precursors that react with melamine to form strong and stable crosslinking networks.
3. They have already demonstrated a wide range of promising physical, chemical and thermal properties, allowing them to be employed for various applications.
4. Melamine-based POPs have large surface areas with microporous structures, making them suitable for post-modification, i.e., as support for stabilized fine metal nanoparticles.

Due to the prevalence of topics on melamine-based POPs and the lack of any review that covers the hotspots and trends in the field, bibliometric analysis was conducted to highlight research directions and provide a visual overview for researchers.

Bibliometric analysis is a quantitative method of analyzing scientific literature and employs statistical approaches. In this quantitative analysis, a large amount of data, extracted from different databases (Scopus, Web of Science and Google scholar), are processed using a set of tools such as R, Python, VOS viewer 1.6.20, and Cite Space. Among these tools, VOS viewer software is considered the most popular tool due to its ease of use and versatility [18,19]. This analysis assists researchers and scientists in understanding the current hot topics and trends in a specific field, as well as future directions. It also depicts countries, entities, notable authors, and the relationships between them. Furthermore, it highlights the most productive journals in the field, allowing researchers to select the best journal to publish their research [20,21,22].

The aim of this review is to provide an in-depth understanding of melamine-based POPs using bibliometric analysis. Overall, 1398 articles were extracted from the Web of Science database (WOS) and they were discussed and visualized via VOS viewer software. The analysis of the literature contains publications numbers, productive authors, countries, institutions, and journals. The collaboration between countries and institutions and keyword co-occurrence are mapped and visualized. Furthermore, successful synthetic strategies for producing melamine-based POPs materials are described and summarized.

## 2. Data Collection

**Figure 2:**
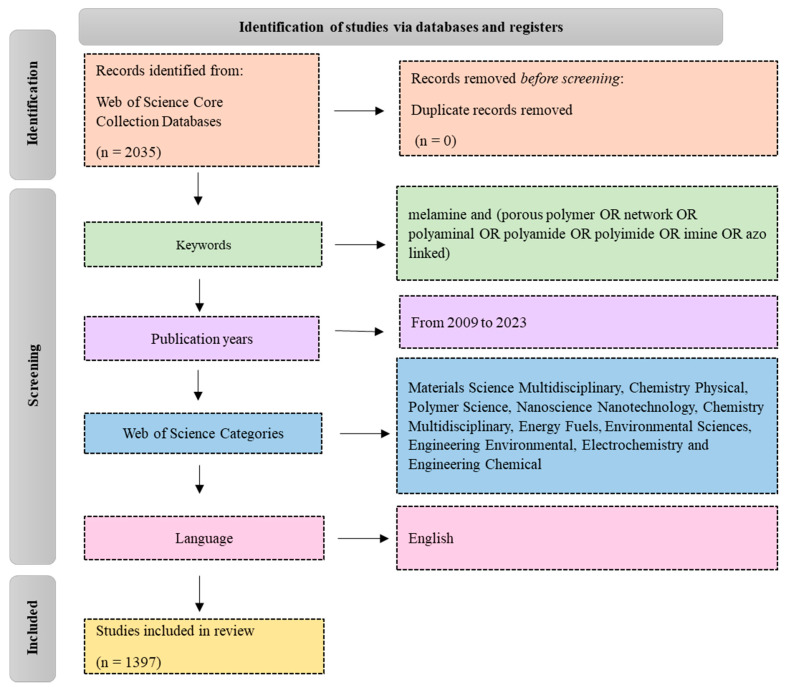
A flow diagram of the review.

This systematic review was conducted in accordance with the Preferred Reporting Items for Systematic Reviews and Meta-Analyses (PRISMA) guidelines. This review paper aims to highlight the most prolific articles that are related to porous polymeric materials based on melamine precures. For this purpose, bibliometric analysis was performed in this study. The research was carried out on 14 July 2024 using the Web of Science Core Collection database. In the initial search on the topic of melamine-based porous polymers, 2035 articles were found and, after applying some filters (publication years, subject area and languages), the final search results amounted to 1397 articles in this research area. The keywords used included (melamine and (porous polymer or network or polyaminal or polyamide or polyimide or imine or azo-linked)). The search data were limited from 2009 to 2023, covering 15 years in literature. To obtain relevant articles, ten Web of Science categories were selected, namely, materials science multidisciplinary, chemistry physical, polymer science, nanoscience nanotechnology, chemistry multidisciplinary, energy fuels, environmental sciences, engineering environmental, electrochemistry, and chemical engineering. Finally, the publication language is set to English, excluding other languages. Figure 2 illustrates document selection and creates a flow diagram for the melamine-based POP topic.

In this review, we employed bibliometric analysis as a quantitative technique to evaluate the data extracted from the Web of Science Core Collection database. This method can be used to depict the landscape of development and the trends in a specific area. VOS viewer 1.6.20, as a common software in bibliometric analysis, was used to visualize and map the search data. Also, Microsoft Excel 365 was employed to evaluate the data, remove duplicate records, and plot the diagrams. The research questions below were addressed using tables, graphs, and maps and discussed, and relationships were found between them.

1. What is the distribution of research articles by years in the field?
2. What are the most relevant authors and journals in the field?
3. What are the most productive countries and institutions in the field?
4. What are the primary research keywords in the field?

This study included indicators of scientific impact, such as the number of total publications and citations and the H-index for authors, institutions, countries, and journals.

### 2.1. Publication Years

**Figure 3:**
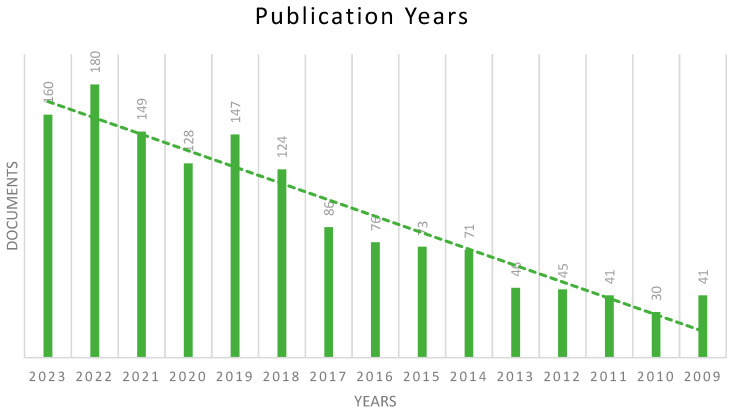
The distribution of articles among publication years (2009–2023).

Figure 3 depicts the distribution of publications in the melamine-based POP field from 2009 to 2023, ranging from 41 to 160 articles. As shown in the graph, there has been a significant increase in the number of published studies in this field, reaching a maximum of 180 articles in 2022. This accounted for 12.9% of the total publications that were published in this period on the topic of melamine-based POPs. Following that, 160 articles were published in 2023, accounting for 11.5% of the total number. The last three years produced the highest publication number compared to the previous years, at 489 articles. This indicates that the research community has become more engaged and productive in this field due to the clear and comprehensive understanding of every aspect of melamine-based POP topics, from the preparation techniques to their beneficial use in various applications.

### 2.2. Document Types and Categories

**Figure 4:**
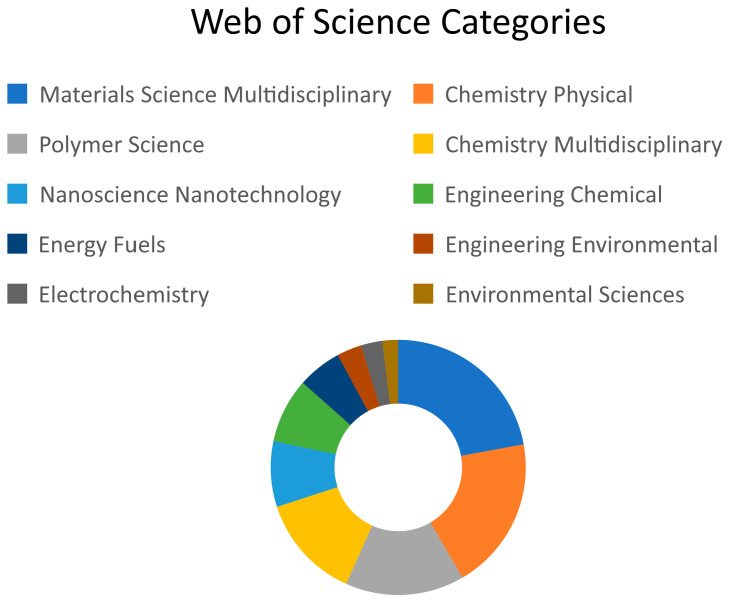
The distribution of article categories.

There are 1397 documents in the field of melamine-based POPs available on the Web of Science database. These documents are categorized into 56 subject areas. The top five Web of Science categories and their corresponding record counts and contribution percentages are materials science multidisciplinary (492) at 35.2%, chemistry physical (434) at 31.1%, polymer science (336) at 24.1%, chemistry multidisciplinary (296) at 21.2%, and nanoscience nanotechnology (185) at 13.2%, as shown in Figure 4. Melamine-based POPs attracted great attention in the materials, chemistry, polymer, and nanotechnology fields, as illustrated by the five top subject areas. In addition, the research directions in this field are gradually migrating to application, involving engineering environmental and environmental science. In general, there is considerable interest in converting raw materials into useful compounds and products and then employing these products in a variety of applications and uses, particularly in environmental fields.

**Figure 5:**
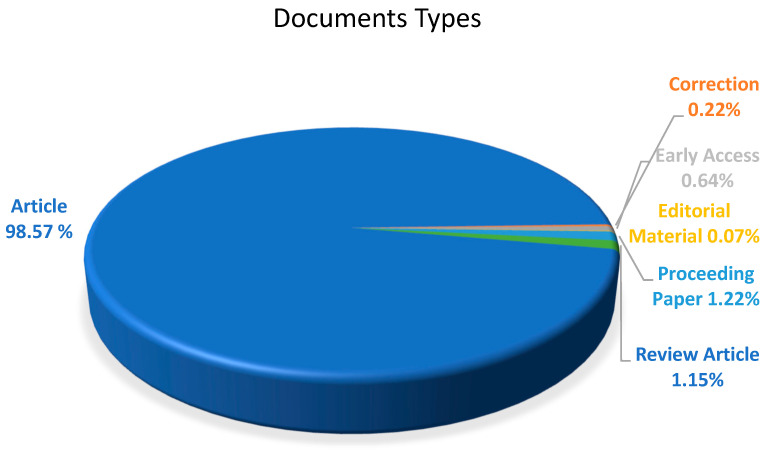
The distribution of document types.

The documents were categorized into six types, as shown in Figure 5, with articles accounting for 98.57% of the documents and 1377 record counts. The remaining 3.3% of contributions were related to proceeding papers (1.22%), review articles (1.15%), early access (0.64%), corrections (0.22%), and editorial material (0.07%) combined. This figure shows us that reviews papers in the field of melamine-based POPs are extremely rare. This encourages researchers in this field to invest more time and effort in creating more thorough reviews that cover every aspect of the topic, from synthesis and design techniques to potential applications.

### 2.3. Active Authors

There are a total of 5351 researchers working in the melamine-based POPs subject area. The top ten productive authors (>13 articles), along with their document number, document contribution, citations, and H-index, are listed in Table 1. The most productive author in the field is Li Qiang with 32 articles, 756 overall citations from these research articles, and an H-index of 17. The second and third authors are Wang Qi and Wang Yong, with publication numbers of 27 and 25 and total citations of 575 and 1020, respectively. Additionally, the first nine authors come from China and the tenth author (Bourbigot, Serge) comes from France. This means that China is the most prominent contributor to the field of melamine-based POPs.

### 2.4. Active Countries and Institutions

The quantitative analysis of countries reveals which countries contribute the most to any specific research area. Overall, 68 countries published 1397 articles on melamine-based POPs. The ten most active countries in the field are listed in the table below according to the number of published articles. As shown in Table 2, the most active country in the field of melamine-based POPs is China, with 863 articles, accounting for 53.03% followed by Germany (92) and India (80).

Moreover, China also had the most total citations (31,305), followed by Germany (3848) and India (1746). This corresponded to the rankings in terms of publication number and H-index.

**Figure 6:**
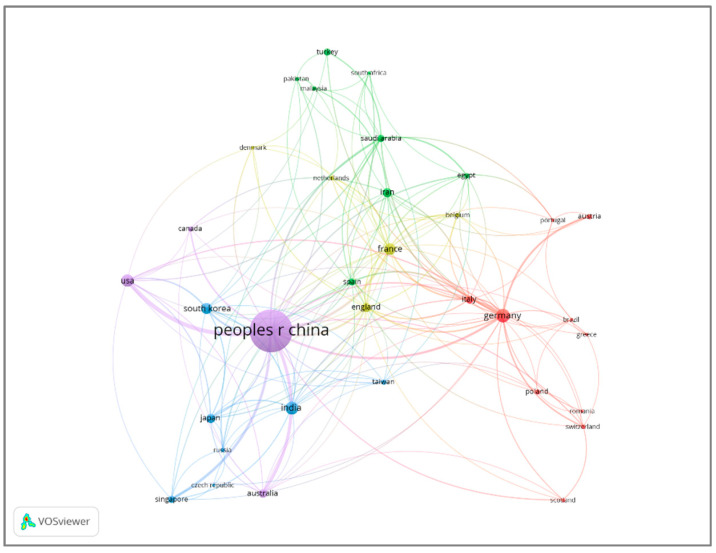
Active countries in the field of melamine-based POPs.

Figure 6 showed a visual representation of countries’ co-occurrence in terms of the topic of melamine-based POPs. The node size means contribution frequencies, while the line thickness indicates a closer collaboration relationship. The different colors represent clusters generated by various groups of countries. As illustrated in Figure 6, China has the biggest circle and the strongest relationship with other countries. The thick line between China and the United States also shows the close connection between the two countries in this research field. Moreover, China and the Kingdom of Saudi Arabia also have a close relationship in this field, which enhances benefit and encourages cooperation to increase their influence and achievements.

The co-occurrence network can help scientists to discover significant organizations and prospective collaborators and determine the number of publications released by institutions. An overall of 1253 institutions were working on the topic. Table 3 shows the top ten institutions; eight of them are from China, and two are from France.

**Figure 7:**
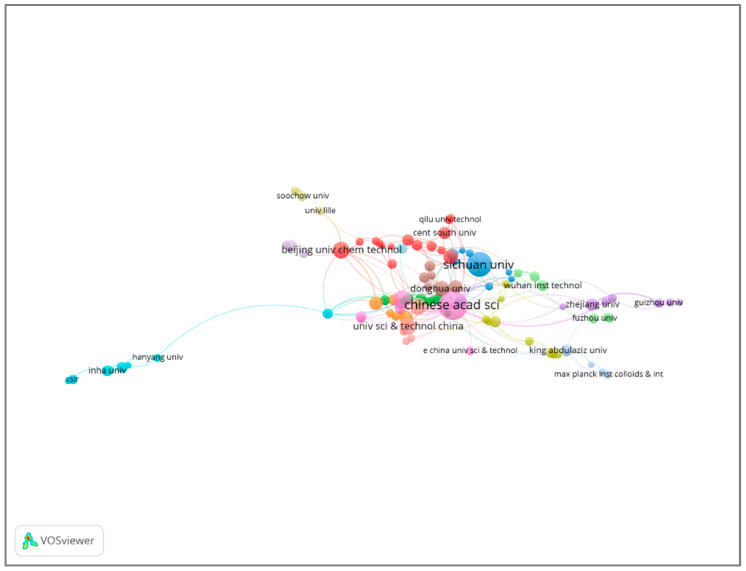
Active institutions in the field of melamine-based POPs.

The Chinese Academy of Sciences and Sichuan University are at the top of the list, with 108 and 60 publications and 4665 and 1902 citations, respectively, followed by the Centre National De La Recherche Scientifique CNRS (42 and 1357). Visual representation was also used to illustrate the interrelationships between universities and research centers, as shown in Figure 7. The size of the node and line reflect the document count per institution, as well as the connection between institutions. It can be concluded that the Chinese university has affiliations with most other organizations, which is consistent with its status as the leading institution in this field and having the most productive researchers.

### 2.5. Active Journals

Qualitative analysis of active journals in the field of melamine-based POPs can help those interested in the field determine which journals are best suited to publish their articles. Table 4 shows the top 10 journals out of the 316 that participate in this domain. The *Journal of Applied Polymer Science* has the most publications on this topic, with 58 articles and 1137 citations. The second and third journals, *Polymer Degradation and Stability* (46, 1926) and *RSC Advances* (45, 919), have nearly identical numbers of articles, but the first has more than twice as many citations as the second. This indicates that research published in the chemistry journal may be more valuable and influential in the field. The journal of *ACS Applied Materials Interfaces* has received the most citations compared to other sources.

### 2.6. Keyword Co-Occurrence

**Figure 8:**
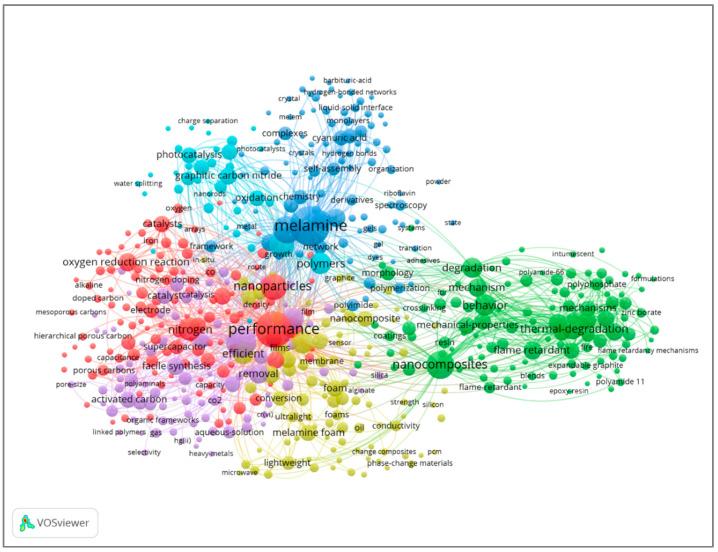
Visual analysis of keyword co-occurrence.

Keyword co-occurrence analysis was performed on data extracted from the Web of Science database. The analysis included 5509 keywords related to melamine-based POPs, 545 of which were repeated at least five times. Table 5 lists the top ten keywords, along with their frequencies. Melamine (236), performance (201), and adsorption (132) were the most frequently employed keywords. Figure 8 depicts the visualization of keyword co-occurrences, with node size representing frequencies and line thickness representing connections between keywords. The co-occurrence map shows that one of the most popular uses for melamine-based POP materials is the adsorption of contaminants, which is followed by thermal degradation methods. Furthermore, the analysis shows that the structural design of melamine-based POP materials takes the form of nanocomposites, composites, nanosheets, networks, and nanoparticles. Particularly, the keywords graphene and polymer appear more frequently, indicating that melamine-based POPs are linked to either inorganic or organic components to produce various materials.

## 3. Synthesis Strategies of Melamine-Based POPs

**Figure 9:**
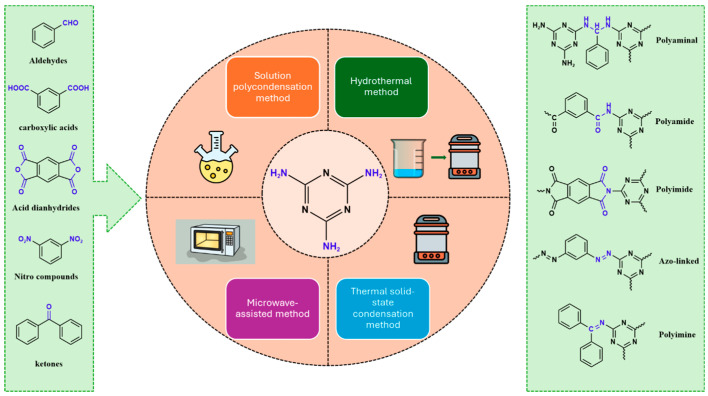
Synthesis strategies of different linkages of melamine-based porous polymers.

Melamine can undergo different reactions and strategies, involving different mechanisms, to yield a variety of polymeric networks. It is possible to categorize melamine-based polymers according to their covalent linkage between melamine units and other organic monomers. As presented in Figure 9, melamine can react with various functional groups such as aldehydes, carboxylic acids, acid dianhydrides, nitro compounds, and ketones to finally yield distinct melamine-based polymers like polyaminal, polyamide, polyimide, azo-linkage, and polyimine networks, respectively [6]. Different approaches and methods are available and widely used for this purpose. In this section, the synthesis strategies applied to different melamine-based polymers are reviewed.

### 3.1. Polyaminal

**Figure 10:**
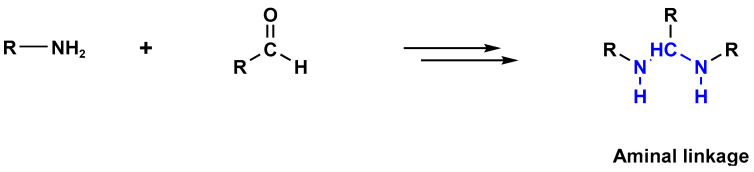
The formation of aminal bonds.

Melamine-based polyaminal networks have several advantages, including increased nitrogen content, hierarchical porous structures, and easy fabrication via the one-pot catalyst-free polycondensation of amine and aldehyde building blocks [23]. Polyaminal networks were frequently used for gas capture and conversion [24], toxic metal elimination [25], and heterogeneous catalytic reactions [26,27,28]. Two steps are involved in the formation of the aminal bonds (-NH-C-NH-). When melamine and aldehyde monomers react, a Schiff-base condensation reaction occurs in the first stage. An imine bond, also known as a Schiff base, was created when the lone pair of amine groups joined the carbonyl carbon. If the amino group is not a potent nucleophile, the second step does not occur. The triazine unit in melamine frequently plays a role in raising the basicity of the linked primary amino groups, enabling them to interact with the Schiff base that has been formed and form stable aminal bonds (Figure 10) [29,30,31]. The literature describes two synthetic strategies used to generate melamine-based polyaminal networks: the traditional one-step polycondensation approach [32] and the microwave-assisted approach [14].

In 2009, the first generation of melamine-based polyaminals was produced using the traditional one-pot polycondensation technique, which is still widely used today [30,33,34,35,36,37,38,39,40,41]

Ibrahim et al. investigated the impact of the benzene rings on the porous structure of the polyaminals as well as on their adsorption capacities. A polyaminal (PAN-NA) network was polymerized through conventional one-step polycondensation between melamine and commercially available 1-naphthaldehyde moieties without utilizing any catalyst. The monomers were dissolved in a dimethyl sulfoxide (DMSO) solvent and allowed to react in a round-bottom flask connected to water reflux and a magnetic stirrer. The reaction was performed at 175 °C under an inert atmosphere for three days. The PAN-NA generated was found to have an 85% reaction yield, a 607.5 m^2^/g specific surface area, and ultra-microporous properties. With an adsorption capacity of 100.7 mg/g, the polyaminal is selective toward the lead ions. Also, the polymer demonstrated good CO_2_ uptake, with 67.9 cm^3^/g at 273 K and 1 bar [40]. Extending this work, Alharthi and colleagues fabricated analogous melamine-based polyaminals with anthracene moieties. To prepare this polymer, the same procedure was followed, but 9-anthracenecarboxaldehyde was used instead of 1-naphthaldehyde. The prepared polymer displayed a BET surface area of 230.4 m^2^/g, a mesoporous structure, and carbon dioxide capture of 30.1 cm^3^/g. According to the author, in comparison to PAN-NA, the polymer shows a lower surface area and thus CO_2_ adsorption capacity. This could be explained by the incomplete condensation reaction that resulted from the anthracene carboxaldehyde monomer’s steric hindrance, which reduced crosslinking and produced few micro/mesopore channels [41].

**Figure 11:**
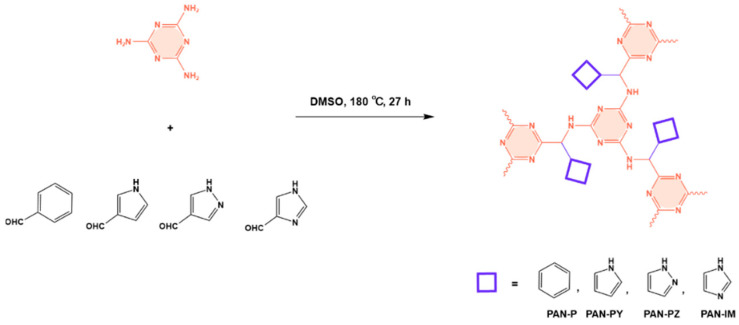
Structure and synthesis schematics of polyaminals for CO_2_ capture.

Shen et al. developed a novel melamine-based porous polyaminal with biphenyl and bipyridyl groups inside the polymer networks. In this work, melamine and aldehydes were mixed and dissolved in dimethyl sulfoxide and an acetic acid solution was added to them. The mixture was subjected to a hydrothermal reactor at 135 °C for three days. The N-rich polyaminal exhibits a loose and porous structure and shows a BET surface area of 330 m^2^/g. The polyaminal network was further used as support to coordinate with Pd(II) salt and was utilized for heterogeneous catalysis [42]. Liu et al. investigates the synthesis of melamine-based POP networks via the polycondensation reaction method. Under reflux and an inert atmosphere, melamine and 4-allyloxy benzaldehyde precursors were reacted at 180 °C for 72 h. The polyaminal (MPOP-4A) produced was washed several times and dried to generate a 75% product yield, which was then kept for further modification. MPOP-4A exhibited a specific surface area of 557 m^2^/g and a mesoporous structure. The polyamial with abundant active sites (nitrogen functionality and H-bonding sites) was applied as an effective heterogeneous catalyst for carbon dioxide cycloaddition [43]. More recently, Liu and his colleagues reported the fabrication of four polyaminals through Schiff-base chemistry (Figure 11). The polyaminals are constructed from melamine with benzaldehyde (PAN-P), pyrrole-3-carbaldehyde (PAN-PY), pyrazole-4-carbaldehyde (PAN-PZ), and Imidazole-4-carbaldehyde (PAN-IM) sub-units. In this approach, a specific amount of melamine, with aldehyde precursors, is mixed in a DMSO solvent in a round-bottom flask connected to water reflux with nitrogen protection at 180 °C and allowed to react for three days. The four polyaminals showed a high specific surface area, ranging from 321 to 562.7 m^2^/g, with hierarchical porous skeletons. Polyaminals with high N-contents and H-bond donor sites were utilized for carbon dioxide capture and conversion. At 273 K, PAN-P exhibits the largest CO_2_ adsorption capacity, measuring 73.5 cm^3^/g. With a 93% yield and 99% selectivity, PAN-IM exhibits the greatest catalytic activity [44].

It is important to note that polyaminal porous polymers with high BET surface areas are produced by this method. Nevertheless, the condensation reaction only yields a 50–66% product and takes a long time to complete (three days) [45].

Microwave-assisted techniques save energy, convert materials more quickly, improve the quality of the product, and are becoming widely used in the manufacturing of inorganic as well as organic materials [46,47].

Yang and his colleagues apply microwave irradiation to synthesize melamine-based polyaminal networks as an alternative to conventional heating. Melamine was reacted with terephthalaldeyde at various molar ratios and a constant amount of dimethyl sulfoxide solvent to produce SNW_A–G_ networks. In the experiment, the reaction mixture was added into a round-bottom flask, which was connected to a magnetic stirrer and thermometer probe, and it was allowed to condense for four hours in a microwave oven set at 180 °C under a nitrogen atmosphere. This method generates polyaminals with a high yield of up to 90% and moderate- to low-surface-area BETs, with surface areas ranging from 115 to 301 m^2^/g. The adsorption capacity of heavy metals on the synthesized polyaminal was studied, and it was found that a soupier adsorption performance was observed toward mercury ions, reaching 1172 mg/g [48]. In a similar context, Zhang et al. synthesized an identical polyaminal by using the same method and starting materials, as previously mentioned. The experiment was conducted under microwave heating for 6 h, resulting in a reaction yield of more than 60% and a specific surface area of 476 m^2^/g. The as-prepared polymer displays exceptionally high reactivity to nitroaromatic explosives [49].

Recently, Sandín et al. utilized microwave irradiation to fabricate three melamine-based polyaminal networks. m-Phthalaldehyde, 2,6-pyridinedicarboxaldehyde, and 2,5-thiophenedicarboxaldehyde moieties were selected to be reacted with melamine under nitrogen protection and 180 °C. The duration of the reaction decreased to 1.5–4 h, and the final product yield increased by more than 80%. Nevertheless, these networks showed a smaller specific surface area of 34–424 m^2^/g [50].

The papers reviewed can be concluded that applying microwave heating to synthesize melamine-based porous polymers surpasses the disadvantages of conventional heating through improving product yield to 90% and reducing the reaction period from three days to 1.5–6 h. However, it produces polymeric networks with relatively moderate to minimal specific surface areas. The fact that the fast condensation reaction between melamine and aldehyde building blocks, performed using microwave irradiation, decreases the degree of crosslinking between monomers and develops terminated amine groups that create H-bonding among MA-based molecules, lowering the porous properties and BET surface area [51,52,53,54,55,56].

Polyaminals are the most reported melamine-based POP materials in literature. The majority are designed and synthesized using a one-pot polycondensation solution-based method. Table 6 below summarizes most melamine-based polyaminals, including their aldehyde building blocks and CO_2_ uptake.

The CO_2_ uptake capacity of melamine-based porous organic polymers (POPs) is strongly influenced by their specific surface area, pore structure, and functional group composition. Generally, materials with higher S(BET) provide more accessible adsorption sites, enhancing CO_2_ capture [51].

### 3.2. Polyamide

Among the first engineering thermoplastics, polyamides are well known for their exceptional mechanical properties, strong intermolecular interactions, and high chemical resistance. These properties are what make polyamides so valuable in the automotive and aerospace industries.

**Figure 12:**
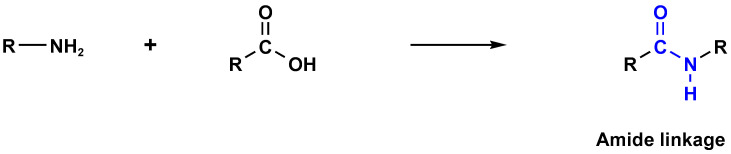
The formation of an amide bond.

Melamine-based polyamides are usually produced through one-pot polyamidation condensation reactions involving melamine and various building units, like carboxylic acids, acyl halides, and acid anhydrides [63,64,65,66,67] (Table 7). In this synthesis, the nucleophilic nitrogen atom of the primary amine group in the melamine molecule attacks the electrophilic carbonyl carbon in the acid monomer, resulting in the removal of H_2_O or HCl molecules as leaving groups and the formation of an amide linkage. A typical reaction mechanism for preparing the polyamide is shown in Figure 12.

**Figure 13:**
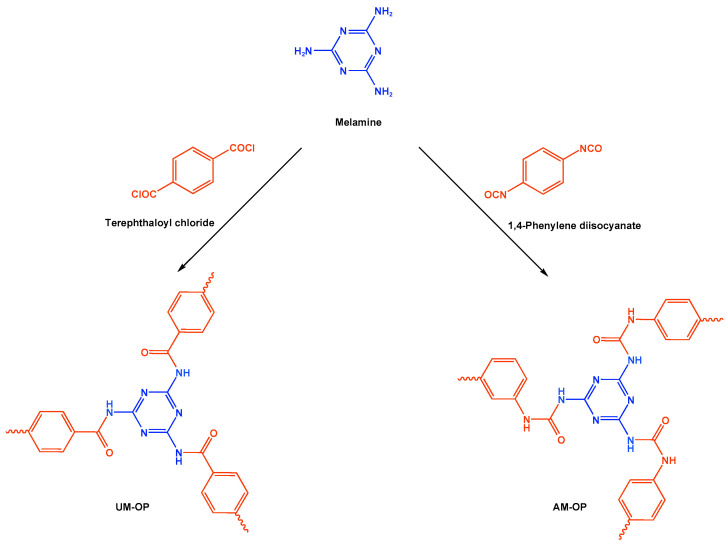
A schematic representation of the synthesis of UM-OP and AM-OP catalysts.

Rehman and coworkers demonstrated a facile polyamidation approach to the fabrication of polyamide networks without using any catalyst. Due to the exothermic behavior of the reaction, the condensation of melamine and diacid chloride precursors (namely terephthaloyl chloride and isophthaloyl chloride) was carried out separately at room temperature in the presence of a dimethylacetamide and methyl-2-pyrrolidone (DMAc/NMP) solvent mixture. Polyamides bearing CO_2_-philic groups from amide linkage and triazene rings showed N-contents of 51 and 45 wt % and exhibited CO_2_ capture values of 8.2 and 3.29 mg/g [68]. Chen et al. developed a series of melamine-based porous polyamides and analogous catalysts using inexpensive and commercially accessible melamine and p-phthalic acid precursors. By conducting various template protocols, synthesized polyamides are obtained with various morphologies and shapes. The diversity of their shape is expected to be effective regarding heterogenous catalytic performance in Suzuki–Miyaura coupling reactions [69]. A study conducted by Zulfiqar and his coworkers showed the effect of two different solvents on the polymerization and porous structure of polyamides. In this study, melamine was reacted with 1,3,5-benzenetricarbonyl trichloride in DMAc/NMP (donated as PA_1_) and in 1,4-dioxane (PA_2_) separately. The monomers were allowed to polymerize in an inert atmosphere for 72 h in a room temperature reaction condition. The synthesized PA_1_ and PA_2_, with reaction yields of 94–90%, were employed for carbon dioxide adsorption [65]. Another study attempted to prepare three polyamides for the removal of mercury ions from wastewater. Through a simple one-pot polyamidation condensation reaction, melamine and three carboxylic acids, specifically phthalic acid, isophthalic acid, and terephthalic acid, were reacted without the use of a catalyst at 180 °C and under atmospheric pressure. The resulting polyamides exhibited BET surface areas ranging from 404 to 521 m^2^/g, microporous and mesoporous structures, and a maximum adsorption capacity of 229.9 mg/g toward mercury ions [70]. In the same context, Shao et al. fabricated a series of polyamides from melamine and four carboxylic acids precursors, namely, o-phthalic acid, m-phthalic acid, p-phthalic acid, and trimesic acid, via a catalyst-free one-pot polyamidation strategy under mild circumstances. In this study, the polymers were prepared through the reaction of melamine with carboxylic acids separately in a dimethyl sulfoxide (DMSO) solvent at 150 °C for three days. The produced polymers had a low to moderate BET surface area ranging from 70 to 521 m^2^/g with nitrogen contents of (13.8–44.9 wt%) and exhibited notable carbon dioxide adsorption up to 2.87 mmol/g at 273 K and 1 bar [71]. Recently, Liu et al. reported the synthesis of melamine-based polymers through the polymerization of melamine with terephthaloyl chloride in a dimethyl sulfoxide solution under N_2_ protection (Figure 13). The resulting polyamide (AM-OP), which had a specific surface area of 24.7 m^2^/g, was employed in a cycloaddition reaction with epoxides to chemically convert CO_2_ into cyclic carbonates [70].

After a review of previous studies, it can be stated that melamine-based polyamides can be synthesized using a variety of techniques, reaction conditions, and building blocks, which has the benefit of yielding polyamides with excellent and desirable properties and characteristics that can be used for significant applications.

**Table 7:** Acid monomers used for the synthesis of polyamides and their applications.

Entry	Materials	Building Block	S(BET) .m^2^/g	Application	Ref.
1	DMAc/NMP	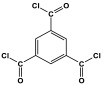	84.5	CO_2_ uptake of 2.99 cm^3^/g	[62]
2	OTPA	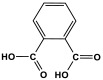	83	1.83 mmol/g	[69]
3	MTPA	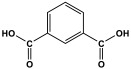	481	2.87 mmol/g	
4	TMPA	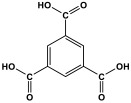	404	2.12 mmol/g	
5	PTPA-3	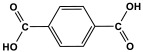	521	2.65 mmol/g	
6	AM-OP	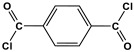	24.72	CO_2_ uptake of 3.23 cm^3^/g	[70]
7	PA2	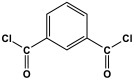	20.18	CO_2_ uptake of 1.7 cm^3^/g	[66]
8	PA6	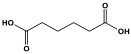	-	-	[71]
9	ME-MA/PAN	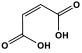	-	For methylene blue removal	[72]

### 3.3. Polyimide

**Figure 14:**
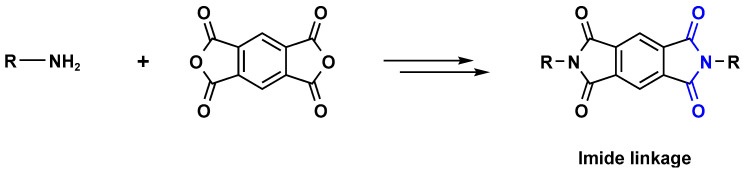
The formation of imide bonds.

Polyimide porous polymers are described as having numerous characteristics, including remarkable redox activity, robust mechanical performance, strong thermal stability, and abundant heteroatoms. Applications such as thermal insulation, photocatalysis, energy storage, and the adsorption of contaminations are attractive for polyimide materials. Moreover, a porous polyimide film is a perfect dielectric solid due to its high dielectric properties [73,74,75,76,77,78,79,80,81,82,83,84,85,86]. The strategy of polyimide synthesis is based on two stages. Firstly, di- or tri-amine and aromatic dianhydride building blocks are adopted to condense inorganic solvents like dimethyl sulfoxide and dimethylacetamide to produce polyamide oligomers. Secondly, the as-prepared polyamide is allowed to polymerize further via a thermal amination procedure, resulting in polyimides with water molecules as byproducts (Figure 14). Famous acid dianhydrides commonly used in polyimide synthesis include pyromellitic dianhydride, benzophenone tetracarboxylic dianhydride, and perylene tetracarboxylic dianhydride. This typical approach generates polyimides with a significant BET surface area and non-crystalline structure [80,81]. Herein, some previous studies on melamine-based polyimides are reviewed. 

**Figure 15:**
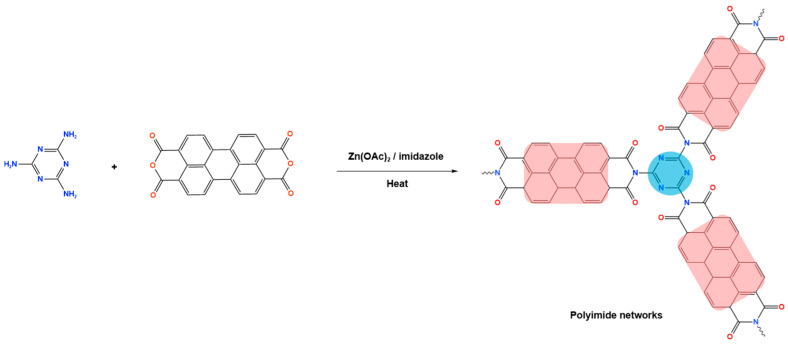
Schematic representation of the synthesis of polyimide networks.

Novel microporous melamine-based polyimides, derived from perylene tetracarboxylic dianhydride and melamine moieties, were designed and synthesized using a simple solution-based approach by Liao et al. [80]. To control the porosity of the synthesized polyimide networks, the reaction was carried out twice, once with the addition of DMSO solvent (as weak solvent template) and once without it. As seen in Figure 15, the dianhydride is combined with the Zn (OAc)_2_/imidazole complex as a Lewis acid catalyst, and it is then agitated at 200 °C under completely dissolved N2 protection. Then, the melamine was dissolved in DMSO and added drop by drop to the mixture and stirred for 24 h. The reaction mixture was kept condensed at 180 °C for a further three days. The polyimides produced show solid yields of 54% (with DMSO) and 47% (without DMSO). Improved porous properties were observed for synthesized polyimide with the presence of DMSO, and this also applied for higher CO_2_ adsorption performances.

Li et al. synthesized two melamine-based polyimides using two readily accessible acid dianhydrides, specifically pyromellitic dianhydride and naphthalene-1,4,5,8-tetracarboxylic dianhydride, as starting materials. Using a solution-based approach, melamine and dianhydride monomers react in N, N-Dimethylformamide for 15 h under an argon atmosphere at 150 °C. After that, the polyamide oligomers were heated up at 330 °C under nitrogen protection for 6 h. The electrochemical performance of the produced polyimides is well suited for sodium-ion batteries [81]. In the same context, a new Fe-chelating melamine-based porous polyimide was synthesized as a heterogeneous photocatalyst by Ding et al. [79]. Melamine with naphthalene tetracarboxylic dianhydride was polymerized in N-methylpyrrolidone under argon protection for 2 days [79]. This reaction produces polyamide acid oligomers with terminated anhydride that are joined with amino groups of melamine under a thermal amination process to finally produce a polyimide network. The as-prepared melamine-based polyimide was incorporated with iron ions via a chelation reaction, and it was employed as catalyst for inducing the free radical polymerization reaction. Polyimides can also be fabricated using the solvothermal method as illustrated by Zhou [82]. This work reported the design of newly created imide-based polymer nanosheets as heterogeneous photocatalysts for effective oxygen activation, driven by visible light. In an autoclave, perylene tetracarboxylic dianhydride was mixed with N, N-Dimethylformamide and ethanediol solvent mixture, and melamine was added. After that, the autoclave was put in the oven with the polymerization parameters set for five days at 200 °C.

For the first time, Kong and coworkers described the production of melamine-based polyimide using the thermal polycondensation process of the salt monomers [83]. Initially, pyromellitic dianhydride underwent an alcoholysis process to obtain pyromellitic acid diethyl esters. The latter was combined with melamine and distributed throughout ethanol, and then the solvent evaporated. The thermal solid-state copolymerization of the salt monomers was carried out at 275 °C in four hours to produce polyimide with a 64.7% reaction yield. Liu and his groups reported the synthesis of a series of polyimide aerogels from oxydiphthalic anhydride and hybrid diamines, namely, oxydianiline and dimethylbenzidine precursors [84]. During synthesis, the diamines and dianhydride are reacted in N-methylpyrrolidinone solvent to form linear anhydride-terminated oligomers. Then, melamine was added as a crosslinker to the mixture and underwent chemical amination reaction to form melamine-crosslinked polyimide aerogels. The use of melamine has proven to be a successful approach for the crosslinking of linear polyimide. The fabricated crosslinked polyimide aerogels were found to possess low shrinking, ultra-low densities, minimum thermal conductivity, and significant thermal stability. Melamine was also used as an additive to polyamide acid precursors, which were then subjected to a thermal imidization process to form melamine/polyimide film [85].

Depending on its intended role and function, melamine can be added to dianhydride in two different ways during the synthesis of polyimide materials, being used as either an affordable crosslinker with already formed linear polyamide oligomers to produce crosslinked polyimides, or as a monomer in the main chain of polyimides for constructing 3D network architectures. Table 8 below summarizes most of the reported melamine-based polyimides, including the dianhydride building blocks and applications.

### 3.4. Azo-Linkage

**Figure 16:**
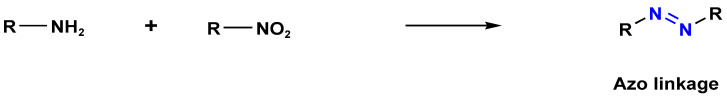
The formation of azo-linkage bonds.

Melamine-based azo-porous polymer networks with (N=N) linkages exhibit encouraging potential applications, including data storage, microdevices, sensors, gas uptake, and energy storage. These networks exhibit a variety of interesting properties, including enhanced thermal stability, abundant N-content, photo chromophore characteristics, and intriguing porous and structural features [16,87,88,89] as shown in Table 9. Azo-linked materials can be created by oxidatively homocoupling aromatic amine compounds and reductively homocoupling aromatic nitro compounds, or by heterocoupling aromatic nitro and amine moieties (Figure 16) [90,91].

Maroufi et al. conducted a study on the synthesis of flame-retardant azo-linked copolymers from melamine and aniline building blocks via a chemical oxidative copolymerization approach [92]. In an acidic medium, monomers were allowed to react with ammonium peroxydisulfate as an oxidizing agent, and the solution was maintained at 0–4 °C under Ar protection for four hours.

**Figure 17:**
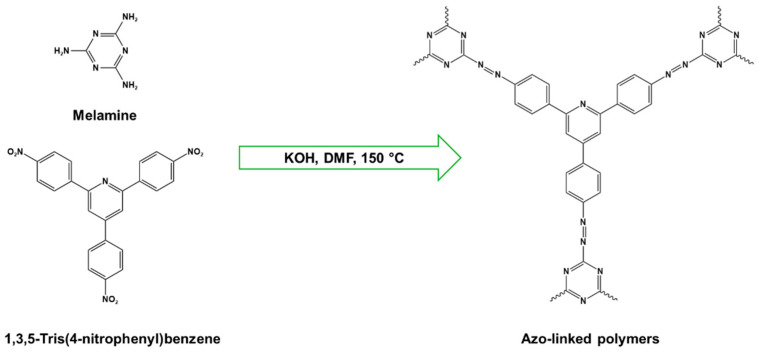
Synthetic route of azo-linked networks.

Prakash et al. synthesized azo-linked porous materials via a condensation polymerization method. In this procedure, melamine with the as-prepared 2,4,6-tris(4-nitrophenyl) pyridine monomer is dissolved in an anhydrous dimethyl formamide solvent and allowed to polymerize in a round-bottom flask fitted with a thermocouple and a magnetic stirrer and connected to reflux system. Then, potassium hydroxide was added to the mixture and the reaction was kept under a nitrogen atmosphere at 106 °C for 24 h. The synthesized materials demonstrated exceptional thermal stability and a capsule-like shape, making them suitable for solar fuel cell uses [93]. In the same context, Ma and his coworker applied the previous technique to generate azo-linked porous polymers as nitrogen-containing precursors to prepped N-doped microporous carbon materials for gas capture (Figure 17). Melamine and 1,3,5-Tris(4-nitrophenyl)benzene building blocks were used to create the azo-linked polymers by adjusting the molar ratios with solid yields of 27.1 and 45.9% [94]. More recently, Youm et al. synthesized a new melamine-based azo-linkage network by the catalyst-free direct coupling of 5,10,15,20-tetrakis(4-nitrophenyl) porphyrin and melamine under basic conditions. The azo-linked network displayed a BET surface area of 630.76 mg^2^/g and reaction yield of 56% and was employed as a catalyst for carbon dioxide conversion [95].

### 3.5. Polyimine

**Figure 18:**
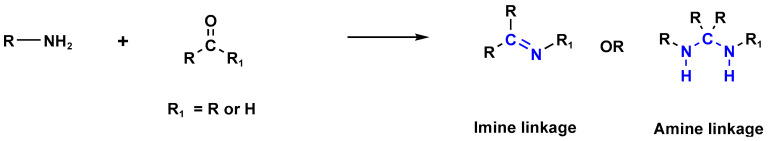
The formation of imine bonds.

Synthesis strategies relating to melamine-based polyimine are like those described for polyaminals through the usage of the multialdehydes and amine building units under similar condensation polymerization conditions (Figure 18). In many cases, polyimine could be produced with crystalline morphology. According to the symmetrical degree of the monomers, the final products could have amorphous or crystalline morphologies [97]. Amorphous POPs were frequently produced by asymmetric compounds containing multiple amines and aldehydes, while crystalline COFs materials were produced by corresponding C3 symmetric monomers.

Chu and coworkers reported a series of semicrystalline imine-based conjugated polymer networks produced via the green Schiff-base thermopolymerization approach without the use of solvent. Based on self-templated methods, a typical process involves the use of NaCl/LiCl mixed salts as a building template with the monomers, namely, melamine and terephthalaldehyde. At 250 °C, the mixture was placed in an autoclave and allowed to react in for 6 h under a nitrogen atmosphere. The porous materials showed a BET surface area of 20–50 m^2^/g and demonstrated promise for the photocatalytic oxidative coupling of amines to imines, reaching 97% conversion of benzylamine in 8 h [98].

Zhao et al. developed melamine-based polyimine porous polymers using the same starting materials as Chu work but working with a hydrothermal synthetic strategy. In this synthetic procedure, a solution of melamine, 4-phthalaldehyde, and dimethyl sulfoxide was prepared and mixed with acetic acid as the catalyst. Subsequently, the reaction mixture was transferred into a reaction kettle and heated to 160 °C for two days. Polyimine with a BET surface area of 231.8 m^2^/g exhibited an adsorption capacity of 2018.1 mg/g toward Hg cations [99]. Alternatively, for the first time, Lin and colleagues reported on the utilization of a mechanochemical strategy, specifically reaction milling, to fabricate POP materials without using organic solvents [100]. Under an Ar atmosphere, milling balls were used to mix the reactants of melamine, terephthalaldehyde, and phytic acid for three hours in a milling jar. The resulting polymer contained a mesoporous structure and showed outstanding electrocatalytic efficiency for the oxygen evolution reaction.

While the solvothermal approach requires several days and needs to be heated at higher temperatures (around 120 °C), reaction milling can drastically reduce the reaction time to 3 h and apply mild reaction conditions, avoiding the requirement for hazardous solvents.

Polyimines could also be produced by using ketones as starting materials instead of dialdehyde precursors in an alternative synthetic pathway [101,102] (Table 10). First, aromatic molecules and acyl halides were used to prepare the two-dimensional ketone-based precursor polymers via the Friedel–Crafts acylation reaction [101,102,103]. Then, to initiate the condensation reaction with the ketone-based precursor polymers, melamine was used as an external crosslinker by post-functionalization. This synthesis method allows for the adoption of a variety of affordable aromatic building blocks in an easy one-pot reaction. The high surface area, abundant porosity, and stable frameworks of the resulting materials make them promising materials for a namouras applications. Wang et al. created melamine-based POPs by using a unique synthesis method based on Schiff-base chemistry. In this work, AlCl_3_ was used as a catalyst in the Friedel–Crafts acylation reaction between tetraphenyl ethylene and benzoyl chloride in dichloroethane solvent. Then, to create a type of novel functionalized POP, melamine was dissolved in DMSO and added to the ketone-functionalized polymer as a rigid external crosslinker. With a combination of imine and secondary amine moieties, the resulting melamine-based polymer demonstrated a high BET surface area of 645 m^2^/g, a distinct hierarchical porosity, exceptional Hg^2+^ removal of 392 mg/g, and CO_2_ capture of 153 mg/g [104].

In addition, by post-functionalizing ketone-based precursor polymers, Wang et al. were able to create bifunctional melamine-based POPs [103]. By using dianhydrides as external crosslinkers and aromatic monomers in a Friedel–Crafts acylation reaction, the precursor polymers were generated. Then, to create bifunctional (imine and amide linkers) melamine-based POPs, melamine was reacted with precursor polymers using the Schiff-base reaction and one-step amidation polycondensation reaction. The resultant materials exhibited outstanding porous properties and abundant N-content, with high performance for carbon dioxide and mercury removal.

**Figure 19:**
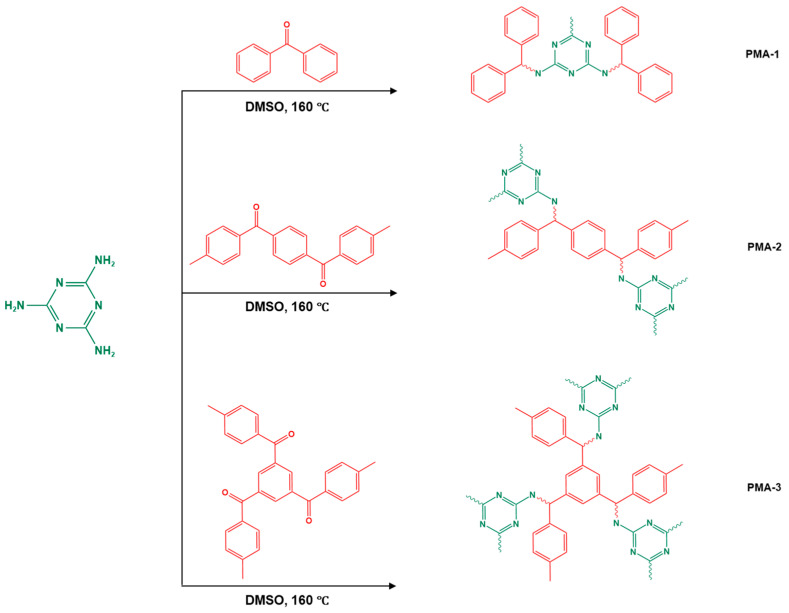
The synthetic procedure of the melamine-based polymer.

Recently, and for the first time, Zhang et al. used small aromatic ketones as building blocks to create melamine-based POPs networks for pollutant adsorption (Figure 19). In this synthesis, the triketones, specifically benzophenone, phenylenebis(*p*-tolylmethanone), and benzene triyltris(*p*-tolylmethanone), reacted with melamine at 160 °C, using dimethyl sulfoxide as the solvent. The proposal mechanism for this polymerization is that dimethyl sulfoxide was initially decomposed to release formaldehyde. Then, it reacted with melamine via a condensation reaction to create intermediate polymers. Final polyimines networks were then created by adding aromatic ketones to the polymer chains in accordance with the Schiff-base reaction. The three porous polymers exhibited higher surface areas, reaching 772 m^2^/g, and showed superior performance in terms of CO_2_ capture (reaching up to 171 mg/g at 273 K and 1 bar) and mercury removal (reaching 702 mg/g at 298 K) [105].

### 3.6. Other Melamine-Based POPs

There are a few reports in the literature on specific types of melamine-based polymers that have not received much attention, like those containing urea linkers [110]. For example, Liu and his group fabricated urea-functionalized melamine-based polymers (UM-OPs) as illustrated in Figure 13. The polymer network was built up from melamine and 1,4-phenylene diisocyanate as starting materials. Using a simple strategy, the monomers were allowed to be polymerized under a N_2_ atmosphere at 100 °C and reflux for one day. The resultant polymer exhibited abundant N sites and -NH- groups with a BET surface area of 46.67 m^2^/g. It showed an outstanding performance in carbon dioxide catalytic cycloaddition [70].

In another context, Ghanbari et al. developed a highly crystalline melamine-based covalent organic framework (mCOF) that was further modified with MOF materials for CO_2_/CH_4_ separation [111]. For this purpose, a one-pot solvothermal method was utilized to prepare a layered-structure mCOF material. Initially, the raw materials of melamine and triethylamine were dissolved in DMF solvent. Cyanuric chloride was added to this solution, and the final solution was put into an autoclave and heated at around 130 °C for one day to finally produce a white product with a BET surface area of 498 m^2^/g.

Various synthetic methods have been employed to develop melamine-based porous organic polymers (POPs), each offering distinct advantages in terms of porosity, chemical stability, and scalability and, in turn, influence adsorption performance. The choice of synthesis route significantly influences the final material properties, making it crucial to compare these methods systematically (Table 11).

A structured comparative analysis of synthetic methods reveals that conventional synthesis significantly influences the porosity, chemical stability, and scalability of melamine-based porous polymeric materials. Conventional condensation, frequently employed in Schiff-base reactions, leads to nitrogen-rich polymers with a high surface area and enhanced adsorption capacity due to strong interactions with polar molecules. This method enables scalable production; however, it often requires long processing times and may result in non-uniform porosity [112].

Microwave-assisted synthesis uses microwave irradiation to speed up the polymerization process and promotes uniform pore formation, improving the crystallinity of the material. However, it tends to produce polymeric networks with relatively lower specific surface areas compared to other methods [113].

Solvothermal synthesis, frequently utilizing high-boiling organic solvents, yields highly porous and chemically stable networks due to controlled polymerization.

Hydrothermal synthesis, typically conducted in aqueous environments, facilitates eco-friendly processing and introduces oxygen-containing functional groups, beneficial for adsorption applications [78].

Systematic studies indicate that polymerization techniques directly influence surface area, nitrogen content, and functional site distribution, areas which dictate adsorption capabilities. For instance, Schiff-base polymerization enhances nitrogen functionalities, improving heavy metal binding, while triazine-based networks exhibit superior gas adsorption due to uniform microporosity [114]. Adsorption pathways primarily involve physisorption in microporous materials and chemisorption in functionalized networks, where amine (-NH_2_) and triazine (-C_3_N_3_) groups play critical roles in selectivity. These findings underscore the necessity of tailoring synthesis parameters to optimize material properties for environmental applications [115].

Melamine-based POPs also undergo post-modifications to enhance selectivity, catalytic efficiency, and stability through functionalization (e.g., amine, sulfonic acid), metalation (e.g., Cu, Fe doping), nanoparticle encapsulation, and structural refinement (e.g., crosslinking, porosity tuning). These strategies improve CO_2_ capture, catalysis, and environmental applications [33,116]. Computational modeling techniques, such as density functional theory (DFT), and molecular simulations have been employed to predict adsorption efficiency and optimize the design of melamine-based POPs [44]. DFT calculations help verify interactions between modified melamine-based POPs and CO_2_, aiding in the development of adsorption mechanisms. These approaches enhance the understanding of adsorption pathways by linking structural modifications to performance, streamlining material development for targeted applications. By integrating experimental data with computational insights, future research can more effectively design POPs with enhanced selectivity and adsorption capacity.

**Table 11:** Comparative summary of synthetic methods for producing melamine-nased POPs.

Method	Porosity and Surface Area	Chemical Stability	Scalability	Advantages	Limitations	References
Conventional condensation	Moderate to high	High	High	Simple, cost-effective	Limited porosity control and long reaction times	[112]
Solvothermal synthesis	High	High	Low (batch process)	High porosity, good crystallinity	Long reaction times	[78]
Hydrothermal synthesis	Moderate to high	High	Medium	Eco-friendly, good functionalization	High temperature requirement	[78]
Microwave-assisted synthesis	Low to moderate	High	High	Fast reaction, energy efficient	Potential heating inhomogeneity	[117]

## 4. Applications for Melamine-Based POPs

Melamine-based porous polymers have garnered significant attention due to their high nitrogen content, tunable porosity, and robust chemical stability. Their diverse applications include CO_2_ capture, water treatment, heterogeneous catalysis, and sensing.

1. CO_2_ Capture

One of the primary applications of melamine-based networks is carbon dioxide capture. This is owing to their high surface area and abundant nitrogen functionalities. [35,55]. Polar functional groups such as -NH and -OH exhibit strong affinity for CO_2_. In these networks, the amine groups derived from melamine contribute to effective CO_2_ adsorption. For example, Zhang et al. synthesized a series of porous polyaminals containing CO_2_-philic groups (-COOH, -OH, and -NO_2_) through a straightforward one-step polymerization process. The incorporation of carboxyl and hydroxyl groups enhances the porous structure, leading to higher S(BET) surface areas and total pore volumes compared to unmodified polymers. This structural improvement significantly boosts CO_2_ capture efficiency and selectivity over N_2_ and CH_4_ gases [60].

2. Water Treatment

Melamine-based POPs with available active sites and amine functionalities have been explored for the removal of organic and inorganic water pollutants such as organic dyes and toxic metals, respectively. The free amines can act as coordinating ligands, binding metal cations very effectively [50]. Seong et al. developed a novel porous organic network by using melamine and vanillin (MVP) as the precursors. The resulting polymer exhibits a high surface area of 745 m^2^/g and a hierarchical pore structure, including both micropores and mesopores. Thanks to the presence of various functional groups such as -NH_2_, -OH, and -OCH_3_, MVP demonstrated effectiveness in removing lead cations [61].

3. Heterogeneous Catalysis

The high nitrogen content and tunable porosity of melamine-based polymers make them excellent supports for catalytic applications. These materials exhibit high thermal stability and surface areas, making them effective as catalysts in various chemical reactions [118]. Chen et al. synthesized a melamine-based polymer-supported Pd catalyst for a Suzuki–Miyaura coupling reaction, demonstrating high conversion rates and recyclability [67].

4. Sensing

Melamine-based polymers are utilized to detect toxic analytes due to their fluorescence and selective adsorption properties. These materials can effectively interact with target analytes through hydrogen bonding, electrostatic interactions, and π–π stacking, making them excellent candidates for sensor development [108]. For example, a study by Zhang et al. developed fluorescent melamine-based polymers that are capable of the sensitive and reusable detection of toxic pesticides [37].

The high surface area and tunable pore structure of, and abundant nitrogen-containing groups in, melamine-based POPs contribute to their effectiveness in adsorbing various pollutants, making them promising materials for a broad range of applications. Future research should focus on improving selectivity and signal transduction mechanisms to expand their practical use in real-world sensing applications.

## 5. Conclusions

This bibliometric analysis provides a comprehensive overview of the progress, research hotspots, and trends in melamine-based porous organic polymers (POPs). The findings highlight the rapid expansion of this field, with significant contributions from materials science, polymer science, and physical chemistry. The increasing interest in melamine-based POPs stems from their tunable porosity, high nitrogen content, and structural versatility, making them promising candidates for adsorption, catalysis, and sensing applications.

Among various applications, CO_2_ capture remains a major research focus due to the strong affinity between CO_2_ and nitrogen-rich frameworks. Studies have shown that melamine-based polymers with microporous structures, hierarchical porosity, and functionalized surfaces exhibit enhanced CO_2_ adsorption capacity. Polymers incorporating additional basic sites (e.g., amines or triazines) and those synthesized via Schiff-base chemistry or polycondensation reactions have demonstrated superior performances. However, challenges remain in optimizing synthesis routes to ensure a balance between surface area, selectivity, and recyclability for real-world applications.

Despite advancements in synthetic strategies including solution-based, hydrothermal, and microwave-assisted methods, scalability remains a critical hurdle. The future direction of research should focus on developing cost-effective, scalable synthesis methods while improving material stability and regeneration efficiency. Additionally, functionalization strategies tailored for specific applications, such as water treatment and heterogeneous catalysis, will be essential for broadening the scope of melamine-based POPs in practical applications. By mapping the evolution of this research area, this study provides insights into effective design principles and emerging directions, guiding future efforts toward the large-scale utilization of melamine-based porous polymers.

## Figures and Tables

**Table 1:** The top 10 active authors.

Rank	Author	Country	Documents	Documents Contribution	Citations	H-Index
1	Li Qiang	China	32	2.291%	756	17
2	Wang Qi	China	27	1.933%	575	15
3	Wang Yong	China	25	1.790%	1020	17
4	Wang Lei	China	18	1.288%	515	10
5	Hu Yuan	China	16	1.145%	598	14
6	Zhang Sheng	China	15	1.074%	476	11
7	Zhang Xiao	China	15	1.074%	466	10
8	Zhang Yong	China	15	1.074%	418	10
9	Li Yue	China	14	1.002%	584	10
10	Bourbigot Serge	France	13	0.931%	480	11

**Table 2:** The top 10 countries in the field.

Rank	Country	Documents	Document Contribution	Citations	H-Index
1	China	863	61.78%	31,305	85
2	Germany	92	6.586%	3848	30
3	India	80	5.727%	1746	23
4	USA	78	5.583%	4306	33
5	France	66	4.724%	1894	22
6	South Korea	61	4.366%	2388	24
7	Iran	44	3.150%	728	18
8	England	42	3.006%	1577	22
9	Japan	40	2.863%	1065	18
10	Australia	35	2.505%	1795	20

**Table 3:** The top 10 institutions in the field.

Rank	Institutions	Country	Documents	Document Contribution	Citations	H-Index
1	Chinese Academy of Sciences	China	108	7.731%	4665	35
2	Sichuan University	China	60	4.295%	1902	22
3	Centre National De La Recherche Scientifique CNRS	France	42	3.006%	1357	18
4	Qingdao University of Science Technology	China	31	2.219%	1070	18
5	University Of Chinese Academy of Sciences Cas	China	31	2.219%	1857	21
6	Beijing University of Chemical Technology	China	28	2.004%	1864	20
7	University Of Science Technology of China Cas	China	26	1.861%	1467	17
8	Donghua University	China	23	1.646%	791	15
9	Central South University	China	22	1.575%	790	13
10	Universite De Lille	France	22	1.575%	591	14

**Table 4:** The top 10 journals.

Rank	Journal	Documents	Documents Contribution	Citations	H-Index	Publisher Name
1	Journal of Applied Polymer Science	58	4.152%	1137	21	Wiley
2	Polymer Degradation and Stability	46	3.293%	1926	28	Elsevier
3	RSC Advances	45	3.221%	919	22	RSC
4	ACS Applied Materials Interfaces	38	2.720%	2166	23	ACS
5	Chemical Engineering Journal	38	2.720%	1920	27	Elsevier
6	Polymers	35	2.505%	499	12	MDPI
7	Journal of Materials Chemistry A	34	2.434%	2051	27	RSC
8	Journal of Physical Chemistry C	25	1.790%	582	16	ACS
9	Carbon	24	1.718%	1687	18	Elsevier
10	Polymers for Advanced Technologies	23	1.646%	386	14	Wiley

**Table 5:** The top 10 most frequent keywords.

Rank	Keywords	Occurrences
1	Melamine	236
2	Performance	201
3	Adsorption	132
4	Nanocomposite	119
5	Networks	114
6	Nanoparticles	104
7	Water	91
8	Nitrogen	80
9	Thermal degradation	69
10	Polymer	70

**Table 6:** Aldehyde monomers used for the synthesis of polyaminals via Schiff-base chemistry and their CO_2_ uptake at 273 K and 1 bar.

Entry	Materials	Building Block	S(BET). m^2^/g	CO_2_ Capture	Ref.
1	PAN-NA	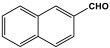	607.5	67.9 cm^3^/g	[40]
2	PAN-AN	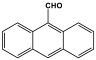	230.4	30.1 cm^3^/g	[41]
3	PAN-PY	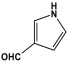	321.0	039. cm^3^/g	[44]
4	PAN-PZ	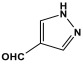	557.4	62.7 cm^3^/g	
5	PAN-IM	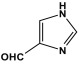	506.5	50.9 cm^3^/g	
6	MA-Py	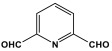	424	-	[50]
7	MBMOP-1	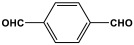	747	57.3 cm^3^/g	[52]
8	MPA-1	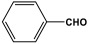	30	38.9 cm^3^/g	[33]
9	MPA-2	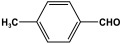	33.5	21.9 cm^3^/g	
10	MOP-1	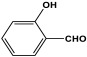	82	33.2 cm^3^/g	[53]
11	MOP-2	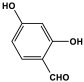	77	29.2 cm^3^/g	
12	MOP-3	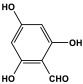	36	58.2 cm^3^/g	
13	PA-M	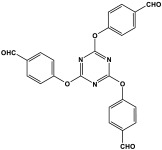	114.29	13.6 cm^3^/g	[35]
14	Bipy-PAN	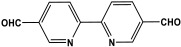	160.7	22.8 cm^3^/g	[54]
15	Py@MA	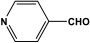	729	43.5 cm^3^/g	[55]
16	Py-Zn@MA	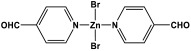	207	29.6 cm^3^/g	
17	Bp-Zn@MA	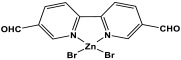	445	26.7 cm^3^/g	[56]
18	PAN-F	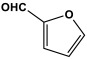	702	75.33 cm^3^/g	[14]
19	PAN-T	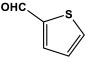	795	50.93 cm^3^/g	
20	PAN-FPP5	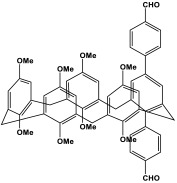	788	63.62 cm^3^/g	[22]
21	PAN-TPDA	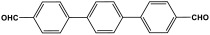	752	58.55 cm^3^/g	
22	TPAMP	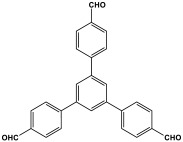	557	40.21 cm^3^/g	[57]
23	TPBMP	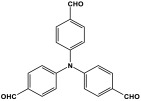	873	85.01 cm^3^/g	
24	TPFM	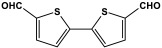	611.3	82.46 cm^3^/g	[58]
25	SNW-2	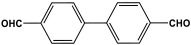	665	75 cm^3^/g	[59]
26	SNW-3	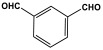	703	93 cm^3^/g	
27	PAN-FP	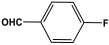	703	66.7 cm^3^/g	[35]
28	PAN-MP	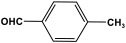	615	59.1 cm^3^/g	
29	PAN-FMP	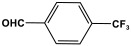	907	74.3 cm^3^/g	
30	PAN-CP	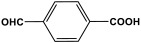	988	67.7 cm^3^/g	[60]
31	PAN-HP	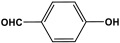	782	68.8 cm^3^/g	
32	PAN-NP	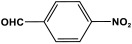	581	52.4 cm^3^/g	
33	TMP-1	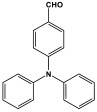	452	52.4 cm^3^/g	[36]
34	TMP-2	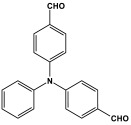	613	69.2 cm^3^/g	
35	MVP	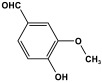	745	-	[61]
36	4A-MBP	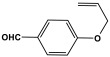	335	-	[62]
37	PA-T1	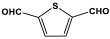	336	18.5 cm^3^/g	[32]
38	PA-T3	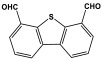	183	7.23 cm^3^/g	
39	PA-F3	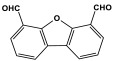	283	9.53 cm^3^/g	
40	PA-T4	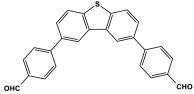	135	25.8 cm^3^/g	
41	PA-F4	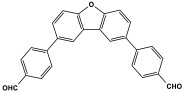	23.8	5.04 cm^3^/g	
42	POPa	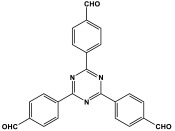	213	-	

**Table 8:** Dianhydride monomers used for the synthesis of polyimides and their applications.

Entry	Materials	Building Block	S(BET) .m^2^/g	Application	Ref.
1	PI-1	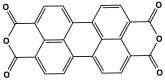	19	CO_2_ uptake of 15.3 cm^3^/g	[80]
2	MPI	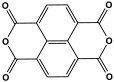	30.6	Photocatalyst for radical polymerization of poly(methyl methacrylate) with molecular weight up to 31.3 × 10^4^ g/mol	[79]
3	R-PA	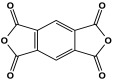		Photocatalyst for selective benzylamine oxidation reactions with % conversion up to 95.2%	[83]
4	MPI-40-10	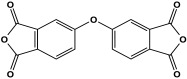	303	-	[84]
5	PI-COF	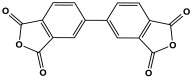	103.50	For O_2_/N_2_ Separation	[86]

**Table 9:** The building blocks used for the synthesis of azo-linked networks and their applications.

Entry	Materials	Building Block	S(BET) .m^2^/g	Application	Ref.
1	PANI	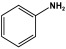	-	Flame retardants	[92]
2	TNPP-M	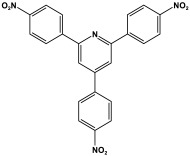	-	For solar fuel cell uses	[93]
3	N-MC-3	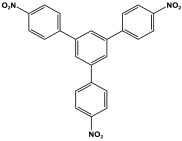	843	Gas adsorption: CO_2_ uptake of 97 cm^3^/g H_2_ uptake of 7.5 cm^3^/g	[94]
4	PBPOP1	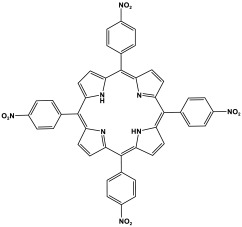	630.76	For CO_2_ capture and conversion	[95]
5	AZO-TPA	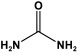	-	For adsorption of iodine and bromine	[96]

**Table 10:** The building blocks used for the synthesis of polyimines and their applications.

Entry	Materials	Building Block	S(BET) .m^2^/g	Application	Ref.
1	MMP	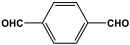	231.8	Hg^2+^ capability of 2018.1 mg/g	[99]
2	Py-TMC-MA	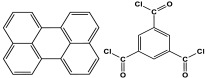	555	CO_2_ uptake of 71.8 cm^3^/g	[101]
3	TPE-OAC-MA	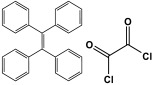	CO_2_ uptake of 73.8 cm^3^/g	CO_2_ uptake of 73.8 cm^3^/g	
4	TPP-TMC-MA	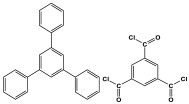	448	CO_2_ uptake of 52.4 cm^3^/g	
5	TPA-TMC-MA	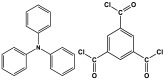	492	CO_2_ uptake of 62.6 cm^3^/g	
6	Py-TC-MA	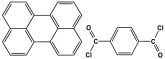	357	CO_2_ uptake of 47.3 cm^3^/g	
7	TPP-TC-MA	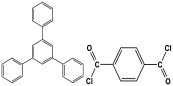	364	CO_2_ uptake of 45.5 cm^3^/g	
8	TPE-TC-MA	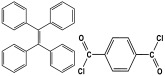	512	CO_2_ uptake of 71.8 cm^3^/g	
9	TPA-TC-MA	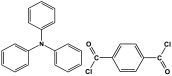	454	CO_2_ uptake of 64.7 cm^3^/g	
10	TPE−PMDA−MA	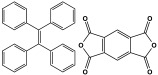	703	CO_2_ uptake of 98.3 cm^3^/g	[103]
11	TPE−PTCDA−MA	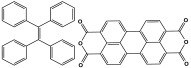	612	CO_2_ uptake of 77.4 cm^3^/g	
12	TPA−PTCDA−MA	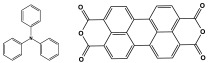	555	CO_2_ uptake of 66.2 cm^3^/g	
13	TPA−PMDA−MA	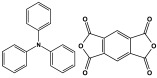	557	CO_2_ uptake of 68.2 cm^3^/g	
14	Py−PTCDA−MA	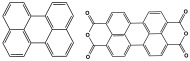	561	CO_2_ uptake of 73.3 cm^3^/g	
15	Py−PMDA−MA	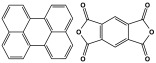	611	CO_2_ uptake of 84 cm^3^/g	
16	PMA-1–8	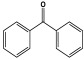	85	Hg^2+^ capability of 462 mg/g	[105]
17	PMA-2–8	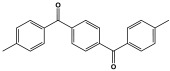	213	Hg^2+^ capability of 501 mg/g	
18	PMA-3–8	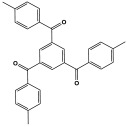	772	Hg^2+^ capability of 702 mg/g	
19	P-MEL-BUT gel	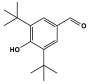	-	Biomaterials for the various biomedical applications	[106]
20	P-MEL-MET gel	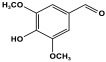	-		
21	PHTCZ-1-MA	PHTCZ-1	613	CO_2_ uptake of 91.6 cm^3^/g	[106]
22	P-2-HPMTT	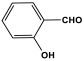	-	-	[107]
23	P-3-HPMTT	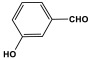	-	-	
24	O-4-HPMTT	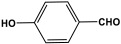	-	-	
25	TDI-co-2-HNMTT	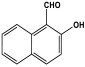	-	Optical Mn(II) sensor	[108]
26	ACP-MEL	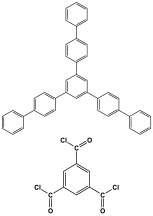	1082	CO_2_ uptake of 134.1 mg/g	[109]
